# PRMT3 methylates HIF-1α to enhance the vascular calcification induced by chronic kidney disease

**DOI:** 10.1186/s10020-023-00759-7

**Published:** 2024-01-10

**Authors:** Guangyu Zhou, Chen Zhang, Hui Peng, Xuesong Su, Qun Huang, Zixia Zhao, Guangyi Zhao

**Affiliations:** 1 Department of Nephrology, Shengjing Hospital of China Medical University, 36# Sanhao Street, Shenyang, China; 2 Department of Anesthesiology, Shengjing Hospital of China Medical University, 36# Sanhao Street, Shenyang, 110004 China

**Keywords:** Chronic Kidney Disease, Vascular calcification, Protein arginine methyltransferase 3, Hypoxia-induced factor 1α, Glycolysis

## Abstract

**Background:**

Medial vascular calcification is commonly identified in chronic kidney disease (CKD) patients and seriously affects the health and life quality of patients. This study aimed to investigate the effects of protein arginine methyltransferase 3 (PRMT3) on vascular calcification induced by CKD.

**Methods:**

A mice model of CKD was established with a two-step diet containing high levels of calcium and phosphorus. Vascular smooth muscle cells (VSMCs) were subjected to β-glycerophosphate (β-GP) treatment to induce the osteogenic differentiation as an in vitro CKD model.

**Results:**

PRMT3 was upregulated in VSMCs of medial artery of CKD mice and β-GP-induced VSMCs. The inhibitor of PRMT3 (SGC707) alleviated the vascular calcification and inhibited the glycolysis of CKD mice. Knockdown of PRMT3 alleviated the β-GP-induced osteogenic transfomation of VSMCs by the repression of glycolysis. Next, PRMT3 interacted with hypoxia-induced factor 1α (HIF-1α), and the knockdown of PRMT3 downregulated the protein expression of HIF-1α by weakening its methylation. Gain of HIF-1α reversed the PRMT3 depletion-induced suppression of osteogenic differentiation and glycolysis of VSMCs.

**Conclusion:**

The inhibitory role of PRMT3 depletion was at least mediated by the regulation of glycolysis upon repressing the methylation of HIF-1α.

## Introduction

Chronic kidney disease (CKD), an irreversible and progressive kidney disease, is linked with the changes of renal structure and degradation of kidney function (Websteret al. 2017). The premature death risk of CKD patient is 5–10 times higher than that of general population, which is largely resulted from the death of cardiovascular diseases (Di Angelantonio et al. 2007; Sarnak et al. 2003; Webster et al. 2017). Notably, medial vascular calcification is commonly identified in CKD patients (Düsinget al. 2021). It causes the decreased compliance of artery walls, leading to the elevation of pulse-wave velocity and systolic pressure. The changes of mechanical and hemodynamic properties contribute to left ventricular hypertrophy, reduced coronary perfusion, and ultimately heart failure as time goes on (Lanzer et al. 2014; Nelson et al. 2020). Moreover, it has been reported that CKD patients exhibit dysregulation of phosphorus and calcium metabolism. High content of serum phosphorus dives vascular calcification, including the transformation of vascular smooth muscle cells (VSMC) into osteoblastic phenotype and extracellular matrix degradation (McCartyet al. 2014, Shanahanet al. 2011). The regulation of osteogenic differentiation of VSMCs may be the key process for the prevention of vascular calcification.

Glycolysis acts as a vital part in glycometabolism and is associated with the osteogenic differentiation of VSMCs (Liet al. 2018). The glycolysis pathway is activated by various calcification factors and overproduces the products of glycolysis including lactate (Yanget al. 2017). Niu et al. have testified that the inhibition of fructose-2,6-biphosphatase 3 (PFKFB3, a key enzyme in glycolysis) could mitigate the glycerophosphate (β-GP)-induced osteogenic differentiation of VSMCs (Niuet al. 2021). The similar effects of glycolysis suppression mediate the repression of vascular calcification could be found in previous studies (Ma et al. 2020; Wang et al. 2022). These researches imply that targeting glycolysis might be a great strategy for the treatment of medial vascular calcification.

Protein arginine methylation is a frequent post-translation modification that is catalyzed by protein arginine methyltransferases (PRMTs) family proteins, which transfer a methyl group to the arginine residues of substrates (Blancet al. 2017). Nine members are identified in PRMTs that could be divided into three types (Blancet al. 2017, Hwanget al. 2021). One of the type I proteins named PRMT3 evoke us much attention. Unlike other members of the PRMT family, PRMT3 exhibits a unique substrate-binding C2H2 zinc finger motif at its N-terminal and a catalytic domain at its C-terminal (Shiet al. 2023). PRMT3 could catalyze the formation of ω-mono- or asymmetric dimethyl arginine, and it mainly locates in cytoplasm rather than distributes in nucleus and cytoplasm (Bachandet al. 2004). Accumulating evidences have demonstrated that the substrates of PRMT3 including RPS2 and HMG1Aα/β (Swiercz et al. 2005; Zou et al. 2007). Lei et al. have reported that PRMT3 facilitates glycolysis by promoting the arginine methylation of lactate dehydrogenase A (LDHA, a key factor in glycolysis) in liver cancer cells (Leiet al. 2022). PRMT3 inhibitor represses the osteogenesis and calcium deposition of mesenchymal stem cells (Minet al. 2019). Based on the vital role of glycolysis and calcification of VSMCs in CKD, it is possible that PRMT3 has the potential on vascular calcification. Furthermore, PRMT3 has been found to drives tumor progressions by the methylation and stabilization of hypoxia-induced factor 1α (HIF-1α) (Liao et al. 2022; Zhang et al. 2021), which could regulate the metabolic switch by upregulating the expression of glycolytic enzymes and flux (Kieranset al. 2021). These studies implied the potential of PRMT3 on vascular calcification might be associated with glycolysis by HIF-1α.

This study aimed to investigate the effects of PRMT3 on vascular calcification. Herein, a CKD model of mice was established, and β-GP-induced VSMCs were used for in vitro assessment. Then, the underlying mechanism was studied.

## Materials and methods

### CKD induction and treatment

Animal experiment was approved by the Medical Ethic Committee of Shengjing Hospital of China Medical University (2022PS997K) following The Guideline for the Care and Use of Laboratory Animals. Eight-week old C57BL/6 mice (male) were housed at 22 ± 1 °C and feed ad libitum for a week of acclimation. They were randomly divided into three groups: Control, CKD, and CKD plus SGC707 (CKD + SGC707). CKD model was established as previously described (Tani et al. 2017; Tóth et al. 2022). Briefly, the mice in CKD and CKD + SGC707 group received a diet with 0.8% phosphate and 0.2% adenine for six weeks and then a diet with 1.8% phosphate and 0.2% adenine for four weeks. Meanwhile, mice in CKD + SGC707 group were intraperitoneal injected with 10 mg/kg SGC707. The dose of SGC707 was chosen according to the previous study (Hoekstraet al. 2019). Mice in control group were fed with 0.8% phosphate for ten weeks. Subsequently, mice were sacrificed, and the serum and aortic tissues were collected for further experiments.

### Biochemical testing

Tissues were mixed with normal saline (1:9, g/v) and then mechanically homogenizing in ice bath. After centrifugation at 430 g for 10 min, the supernatant was collected. Meanwhile, cells resuspended with phosphate buffered saline were subjected to ultrasonication, followed by the collection of cell supernatant. The supernatant of tissues and cells, as well as serum of mice were used for further detection, respectively. The levels of blood urea nitrogen (BUN), creatinine, alkaline phosphatase (ALP), phosphorous, and calcium were detected using the Urea Assay Kit, Creatinine Assay kit, Alkaline Phosphatase Assay kit, Phosphate Assay Kit, and Calcium Assay Kit. All the kits were purchased from Nanjing Jiancheng Bioengineering.

### Histological examination

Paraffin-embedded sections of aorta (5 μm) were deparaffinized in xylene, and rehydrated in an ethanol gradient with distilled water. For the assessment of aortic calcium deposition, the sections were stained with Alizarin Red S reagent (Solarbio, Beijing, China) according to the user’s protocols. For the evaluation of PRMT3 expression, after block with 1% bovine serum albumin for 15 min at room temperature, the sections were incubated with the primary antibody anti-PRMT3 (1:50; Proteintech, Wuhan, China) at 4 °C overnight, followed by the incubation with the secondary antibodies horseradish peroxidase-conjugated goat anti-rabbit IgG (1:500; ThermoFisher Scientific, Pittsburgh, PA, USA) at 37 °C for 60 min. Subsequently, the sections were counterstained with hematoxylin for 3 min. For the measurement of pathological changes, the sections were conducted to hematoxylin and eosin staining.

### Western blot

Total protein was extracted from tissues or cells using the RIPA lysis solution mixed with phenylmethanesulfonyl fluoride (100:1, v/v). The protein concentration was detected with BCA Protein Assay Kit (Solarbio). The protein was separated by 10% sodium dodecyl sulfate polyacrylamide gel electrophoresis and then transferred to polyvinylidene fluoride (Merk Millipore, Billerica, MA, USA). After block with blocking solution (Solarbio), the bands were incubated with the primary antibodies 4 °C overnight and then the secondary antibodies at 37 °C for an hour. The probes were incubated with electrochemiluminescence for 5 min, and the images were captured with a gel imaging system. The primary antibodies were as follows: Anti-alpha smooth muscle actin (α-SMA; 1:20000; Proteintech), anti-osteopontin (OPN; 1:2000; Proteintech), anti-osteocalcin (OCN; 1:2000; ABclonal, Wuhan, China), anti- smooth muscle 22 alpha (SM22α; 1:1000; ABclonal), anti-PRMT3 (1:1000; Proteintech), anti-smooth muscle myosin heavy chain (SMHHC; 1:1000; ABclonal), anti-glucose transporter type 1 (GLUT1; 1:1000; ABclonal), anti-pyruvate kinase M1/2 (PKM2; 1:1000; ABclonal), anti-HIF-1α (1:1000; Affinity Biosciences, Changzhou, China), anti-PFKFB3 (1:1000; ABclonal), anti-lactate dehydrogenase A (LDHA; 1:1000; ABclonal), and anti-GAPDH (1:10000; Proteintech). The secondary antibodies were as follows: Horseradish peroxidase-conjugated goat anti-rabbit IgG (1:3000; Solarbio) and horseradish peroxidase-conjugated goat anti-mouse IgG (1:3000; Solarbio).

### Cell culture and cell calcification model

Human VSMCs were purchased from Procell (Wuhan, China). VSMCs were cultured with F12K medium supplemented with 10% fetal bovine serum at 37 °C in a 5% CO_2_ incubator. VSMCs were conducted to a detection using the short tandem repeat profiling and PCR Mycoplasma Test Kit (Wanleibio, Shenyang, China) to confirm that the cells were not contaminated with other cell lines or mycoplasma contamination. After infected with/without lentivirus for 48 h, cells were treated with 10 mM β-glycerophosphate (β-GP) for the induction of calcification for 1, 3, 7, and 14 d. Then, treatment with β-GP for 7d was selected for further experiments.

### Adenovirus preparation

For the knockdown of PRMT3, short hairpin RNA targeting PRMT3 (shPRMT3) was designed and synthetized by GenScript (Nanjing, China). shPRMT3 or negative control was inserted into pShuttle-CMV-H1. The recombinant vector Ad-shPRMT3 (PRMT3^(−)^) and Ad-negative control shRNA (NC), was confirmed by enzymes digestion and sequence. Next, PRMT3^(−)^ was transfected into HEK293A cells with helper plasmid pAdEasy-1. The virus was collected and filtered using a 0.45-µm filter. Subsequently, VSMCs were infected with the particles with the multiplicity of infection at 100.

For the overexpression of HIF-1α, the cDNA of HIF-1α was amplified and subcloned into pShuttle-CMV. The recombinant vector Ad-HIF-1α (HIF-1α^(+)^), and Ad-Vector (Vec) was transfected into HEK293A cells with pAdEasy-1. The particles were collected as described above, and then co-infected with PRMT3^(−)^ into VSMCs.

### Quantitative RT-PCR

Total RNA was extracted from tissues or cells using the TRIpure Reagent (BioTeke, Beijing, China) and then conducted to cDNA synthesis using the BeyoRT II M-MLV reverse transcriptase (Beyotime, Shanghai, China) in accordance with manufacturers’ instructions. Amplification was performed by employing the 2×Taq PCR MasterMix (Solarbio) in the presence of SYBR Green (Solarbio). Beta-actin was used as the internal control. The primer sequence was shown in Table 1.

**Table 1:** Sequences of primers used in quantitative RT-PCR

Genes	Sequences (forward 5’-3’)	Sequences (reverse 5’-3’)
PRMT3 (*mus*)	CAGAGTGGATGGGCTATTT	CAGCTACAAGGCTGATGGT
PRMT3 (*homo*)	AGCCTTGTAGCAGTGAGT	AAATAAGAGTCTTCGGATC
RUNX2 (*homo*)	CTACTATGGCACTTCGTCAGG	TTCCATCAGCGTCAACACC
GLUT1 (*homo*)	TCATCGCCCAGGTGTTCGG	GTTCTCCTCGTTGCGGTTG
PFKFB3 (*homo*)	GCGGGAGCAGGACAAGTA	CGACAGGCGTCAGTTTCA
PKM2 (*homo*)	GACATTGATTCACCACCCA	GTTCAGACGAGCCACATTC
LDHA (*homo*)	TCGAAGACAAATTGAAGGGAG	GTAACGGAATCGGGCTGAA

### Co-immunoprecipitation (Co-IP)

Protein samples were subjected to immunoprecipitation using a Pierce Co-IP Kit (Pierce, immunoprecipitation, IL, USA) in accordance with the users’ protocols. In brief, anti-PRMT3 or anti-HIF-1α antibodies were covalently linked to an amine-active resin in a column. Next, the samples was added to the column and then incubated at 4 °C overnight. The immunoprecipitate of the protein samples with IgG was employed as a negative control. The co-precipitated protein was further subjected to western blot analysis.

### Statistical analysis

Data was presented with mean and standard deviation of three independent experiments in vitro and six independent experiments in vivo. Statistical analysis was performed using GraphPad Prism 8.0 software (La Jolla, California, America). Comparsion between two groups was performed using Student’s t-test. Differences among multiple groups were compared with one-way ANOVA analysis. Differences with p value < 0.05 were considered statistically significant.

## Results

### PRMT3 is upregulated in thoracic aorta of CKD mice

**Fig. 1:**
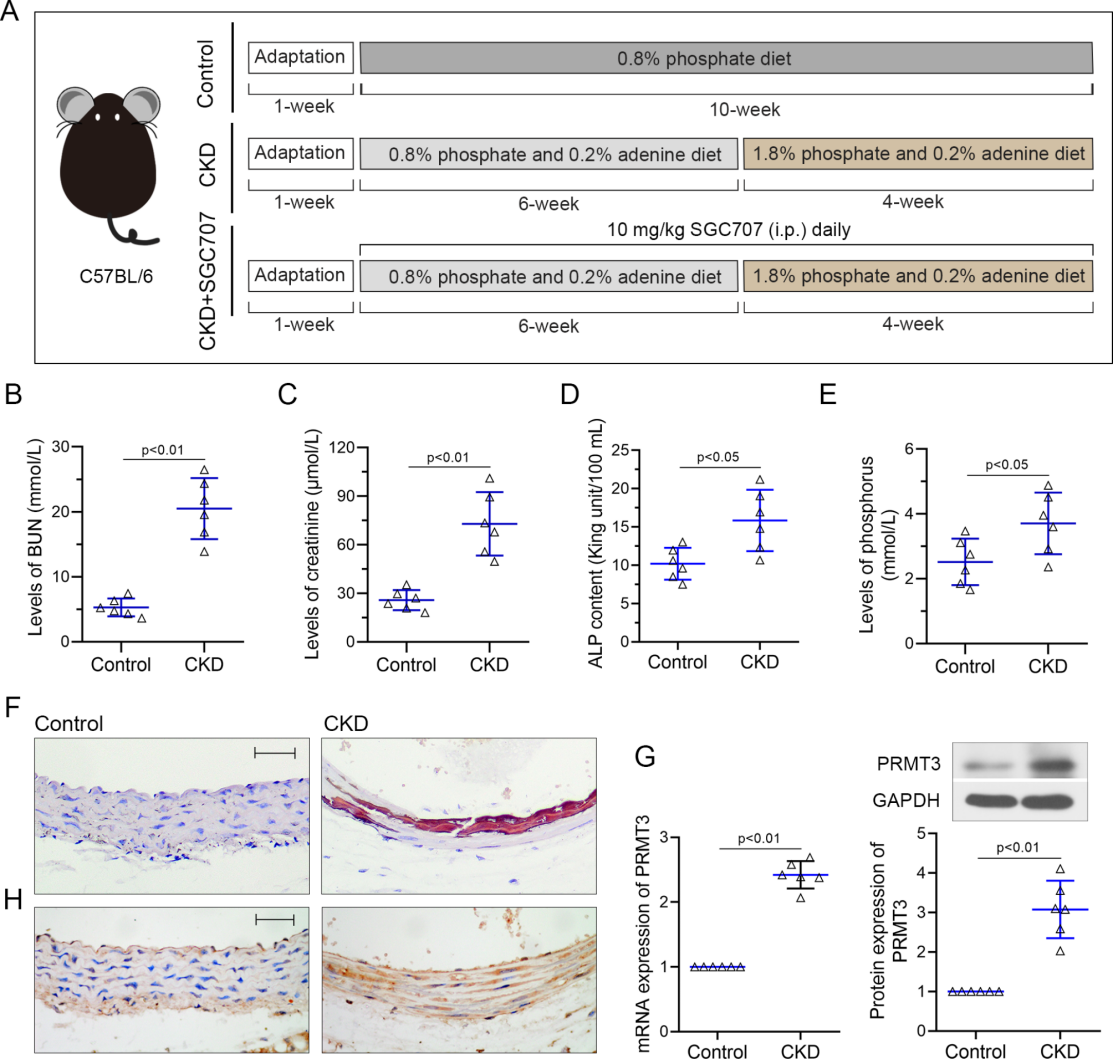
PRMT3 is upregulated in thoracic aorta of CKD mice C57BL/6 mice were received a diet containing 0.8% phosphorus and 0.2% adenine for six weeks, followed by a diet containing 1.8% phosphorus and 0.2% adenine for four weeks. **(A)** Experimental protocol of CKD mouse model. The levels of BUN **(B)**, creatinine **(C)**, ALP **(D)**, and phosphorus **(E)** were elevated in the serum of mice. **(F)** Aorta calcification was evaluated by Alizarin Red S. Scar bar is 50 μm. **(G)** mRNA and protein levels of PRMT3 were detected in aorta of mice. **(H)** PRMT3 expression was assessed by immunohistochemical staining. Scar bar is 50 μm. Data is represented as mean ± SD. p < 0.05, p < 0.01 vs. Control. ALP: alkaline phosphatase; BUN: blood urea nitrogen; CKD: chronic kidney disease; PRMT3: protein arginine methyltransferase 3

To investigate the pathological changes of CKD, a mice model was established with high phosphorus diet as described above (Fig. 1A). As shown in Fig. 1B-E, the levels of BUN, creatinine, ALP, and phosphorus were elevated in the serum of CKD mice, indicating the renal injury and vascular calcification of CKD mice. Alizarin Red S staining confirmed that the aortic sections in CKD mice exhibited severe aortic calcification compared to control (Fig. 1F). Subsequently, the mRNA and protein expression of PRMT3 was detected. The results revealed that PRMT3 was upregulated in aorta of CKD mice (Fig. 1G). The results of immunohistochemical staining further confirmed the upregulation of PRMT3 in VSMCs of medial artery (Fig. 1H).

### PRMT3 inhibitor SGC707 represses the vascular calcification and glycolysis of CKD mice

**Fig. 2:**
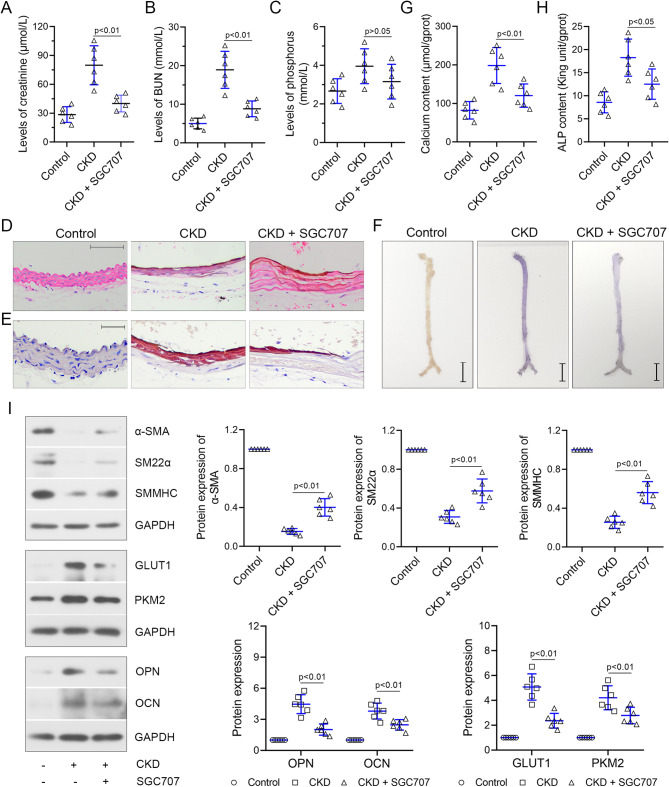
PRMT3 inhibitor SGC707 inhibits CKD-induced vascular calcification in vivo C57BL/6 mice were received a diet containing 0.8% phosphorus and 0.2% adenine for six weeks, followed by a diet containing 1.8% phosphorus and 0.2% adenine for four weeks. Meanwhile, mice were intraperitoneally injected with 10 mg/kg SGC707. The levels of **(A)** creatinine, **(B)** BUN, and **(C)** phosphorus were elevated in the serum of mice, which were reduced by SGC707 treatment. **(D)** Pathological injury was assessed with H&E staining. Scar bar is 100 μm. **(E)** Aorta calcification was evaluated by Alizarin Red S. Scar bar is 50 μm. **(F)** Alizarin Red S-stained aorta. Scar bar is 0.5 cm. **(G)** Calcium content of aorta. **(H)** ALP content. **(I)** Western blot bands of α-SMA, SM22α, SMMHC, OPN, OCN, GLUT1, and PKM2. Data is represented as mean ± SD. p < 0.05, p < 0.01 vs. CKD. ALP: alkaline phosphatase; α-SMA: alpha smooth muscle actin; CKD: chronic kidney disease; GLUT1: glucose transporter type 1; H&E: hematoxylin and eosin; HIF-1α: hypoxia inducible factor 1 subunit alpha; OPN: osteopontin; OCN: osteocalcin; PKM2: pyruvate kinase M1/2; PRMT3: protein arginine methyltransferase 3; SM22α: smooth muscle 22 alpha; SMMHC: smooth muscle myosin heavy chain

The effects of PRMT3 on the CKD-induced renal function and vascular calcification were further explored. SGC707 (PRMT3 inhibitor) attenuated the CKD-induced renal injury of CKD mice, as evidenced by the low levels of creatinine and BUN (Fig. 2A-B). There was a trend that the increased phosphorus content was reduced by SGC707 (Fig. 2C). Then, histological changes were assessed by ematoxylin-eosin and Alizarin Red S staining. Hematoxylin-eosin staining demonstrated that neatly arranged VSMCs and elastic lamellae were shown in control group, whereas disorder, vacuolization, and loss of elastic lamellae and VSMCs were observed in artery of CKD mice (Fig. 2D). SGC707 alleviated the pathological injuries (Fig. 2D). Next, SGC707 reduced the CKD-induced calcium deposition of artery (Fig. 2E-F). The decrease of calcium content in CKD mice with SGC707 treatment echoed the results indicated above (Fig. 2G). Next, SGC707 inhibited the osteogenic differentiation, as evidenced by the reduction of ALP content (Fig. 2H), upregulation of α-SMA, SM22α, SMMHC and the downregulation of OPN and OCN (Fig. 2I). SGC707 decreased the CKD-induced upregulation protein levels of GLUT1 and PKM2 (Fig. 2I), indicating the repressed function in glycolysis.

### PRMT3 is upregulated in the β-GP-induced VSMCs and facilitates the calcification

**Fig. 3:**
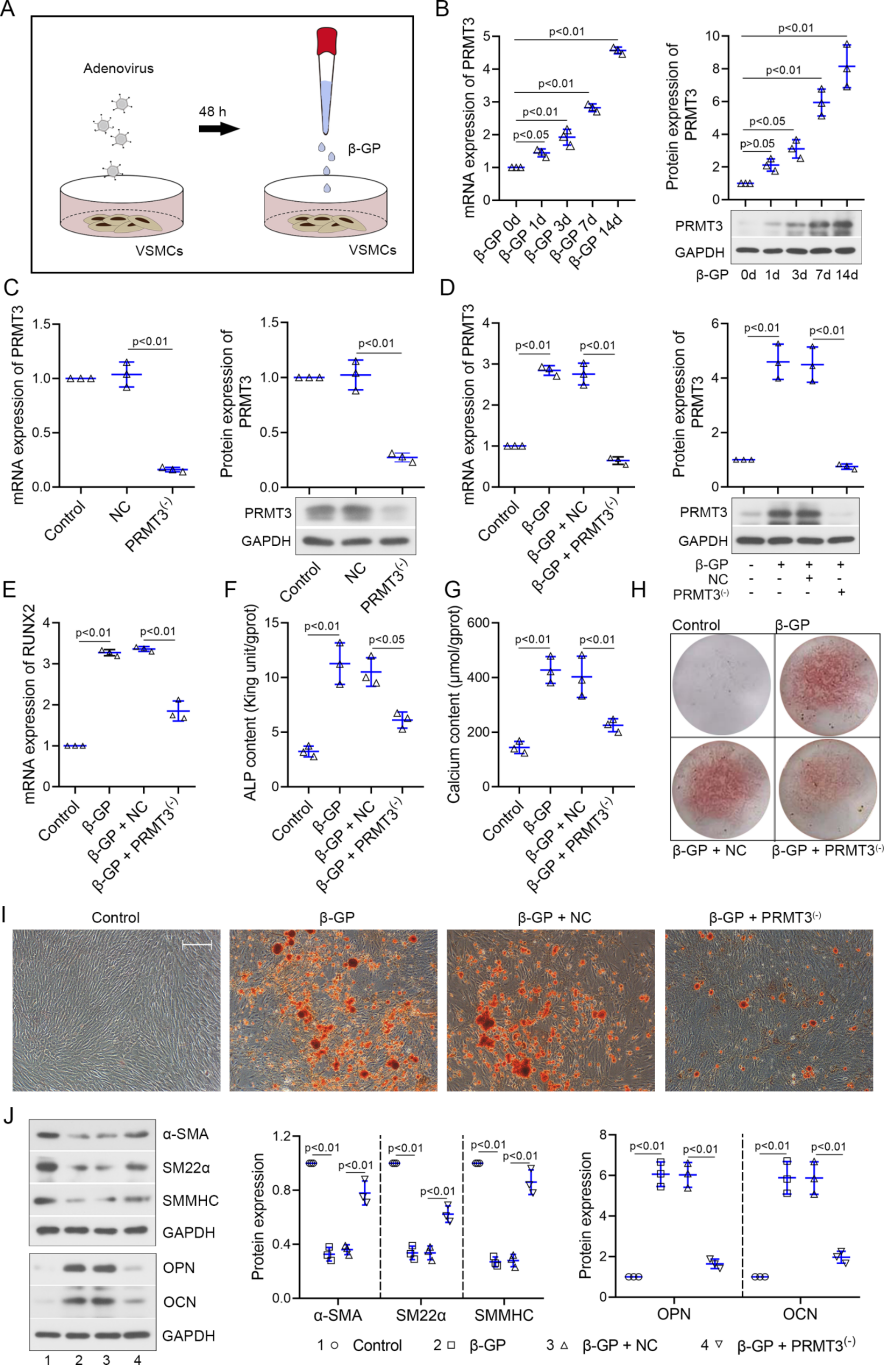
PRMT3 is upregulated in the β-GP-induced VSMCs and facilitates the calcification **(A)** Experimental protocol of VSMCs. **(B)** mRNA and protein expression of PRMT3 in VSMCs incubated with 10 mM β-GP for 1 d. 3 d, 7 d, and 14 d. **(C)** mRNA and protein expression of PRMT3 in VSMCs infected with/without adenovirus Ad-shPRMT3 (shown as PRMT3^(-)^) or Ad-shNC (shown as NC). **(D)** mRNA and protein expression of PRMT3 in VSMCs infected with adenovirus, followed by the incubation with/without 10 mM β-GP. **(E)** mRNA expression of RUNX2. **(F)** ALP and **(G)** calcium content. **(H-I)** Alizarin Red S staining was performed to evaluate the calcification of VSMCs. Scale bar is 100 μm. **(J)** Western blot bands of α-SMA, SM22α, SMMHC, OPN, OCN. Data is represented as mean ± SD. p < 0.05, p < 0.01 vs. β-GP 0 h, control, NC, or β-GP + NC. ALP: alkaline phosphatase; α-SMA: alpha smooth muscle actin; β-GP: β-glycerophosphate; NC: negative control; OPN: osteopontin; OCN: osteocalcin; PRMT3: protein arginine methyltransferase 3; RUNX2: RUNX family transcription factor 2; SM22α: smooth muscle 22 alpha; SMMHC: smooth muscle myosin heavy chain; VSMCs: vascular smooth muscle cells

To deeply investigate the underlying mechanism of the promoted role of PRMT3 in media calcification, a widely used cell model was established with β-GP treatment (Fig. 3A). PRMT3 was upregulated in VSMCs with β-GP in a time-dependent manner (Fig. 3B). mRNA and protein expression of PRMT3 was decreased in VSMCs infected with adenovirus PRMT3^(−)^ compared to VSMCs infected with NC (Fig. 3C). Knockdown of PRMT3 reduced the β-GP-induced upregulation of PRMT3 and osteogenic marker RUNX family transcription factor 2 (RUNX2) (Fig. 3D-E). Then, Loss of PRMT3 weakened the ALP and calcium content of VSMCs in the presence of β-GP (Fig. 3F-G). The similar results were confirmed by Alizarin Red S staining (Fig. 3H-I). PRMT3 silencing elevated the protein levels of α-SMA, SM22α, SMMHC, and decreased the expression of OPN and OCN (Fig. 3J). These results implied that PRMT3 downregulation dampened the calcification of VSMCs induced by β-GP.

### PRMT3 knockdown downregulates HIF-1α and represses the glycolysis of VSMCs induced by β-GP

**Fig. 4:**
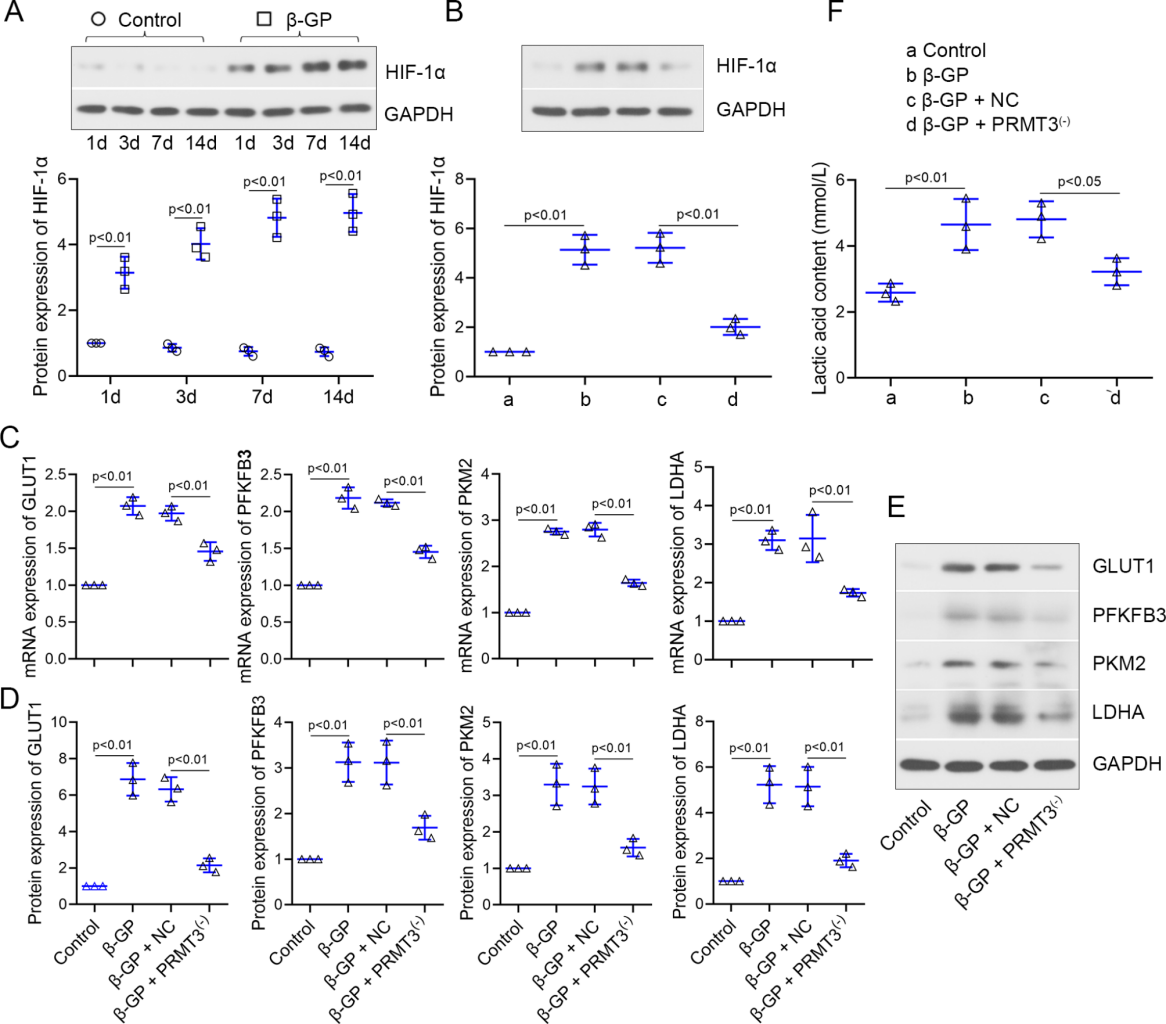
PRMT3 knockdown downregulates HIF-1α and represses the glycolysis in VSMCs induced by β-GP **(A)** Protein expression of HIF-1α in VSMCs incubated with 10 mM β-GP for 1 d, 3 d, 7 d, and 14 d. **(B)** Protein expression of HIF-1α in VSMCs infected with adenovirus Ad-shPRMT3 (shown as PRMT3^(−)^) or Ad-shNC (shown as NC), and then incubated with/without 10 mM β-GP. **(C-E)** mRNA and protein levels of GLUT1, PFKFB3, PKM2, and LDHA in VSMCs. **(F)** The contents of lactic acid. Data is represented as mean ± SD. p < 0.05, p < 0.01 vs. control or β-GP + NC. β-GP: β-glycerophosphate; GLUT1: glucose transporter type 1; HIF-1α: hypoxia inducible factor 1 subunit alpha; LDHA: lactate dehydrogenase A; NC: negative control; PFKFB3: 6-phosphofructo-2-kinase/fructose-2,6-biphosphatase 3; PKM2: pyruvate kinase M1/2; PRMT3: protein arginine methyltransferase 3; VSMCs: vascular smooth muscle cells

HIF-1α could promote the key enzymes in glycolysis, which might act as an essential part in vascular calcification. The role of HIF-1α evoke us much attention. As shown in Fig. 4A, the protein expression of HIF-1α was upregulated in VSMCs with β-GP treatment in a time-dependent manner. Interfering in the expression of PRMT3 reduced the upregulation of HIF-1α protein expression in VSMCs induced by β-GP (Fig. 4B). Then, PRMT3 depletion decreased the β-GP-induced upregulation of GLUT1, PFKFB3, PKM2, and LDHA (Fig. 4C-E). The content of lactic acid was also declined by PRMT3 silencing (Fig. 4F). The data suggested that PRMT3 knockdown repressed the glycolysis in VSMCs induced by β-GP.

### HIF-1α overexpression reversed the PRMT3 knockdown-induced repression of VSMCs calcification and glycolysis in the presence of β-GP

**Fig. 5:**
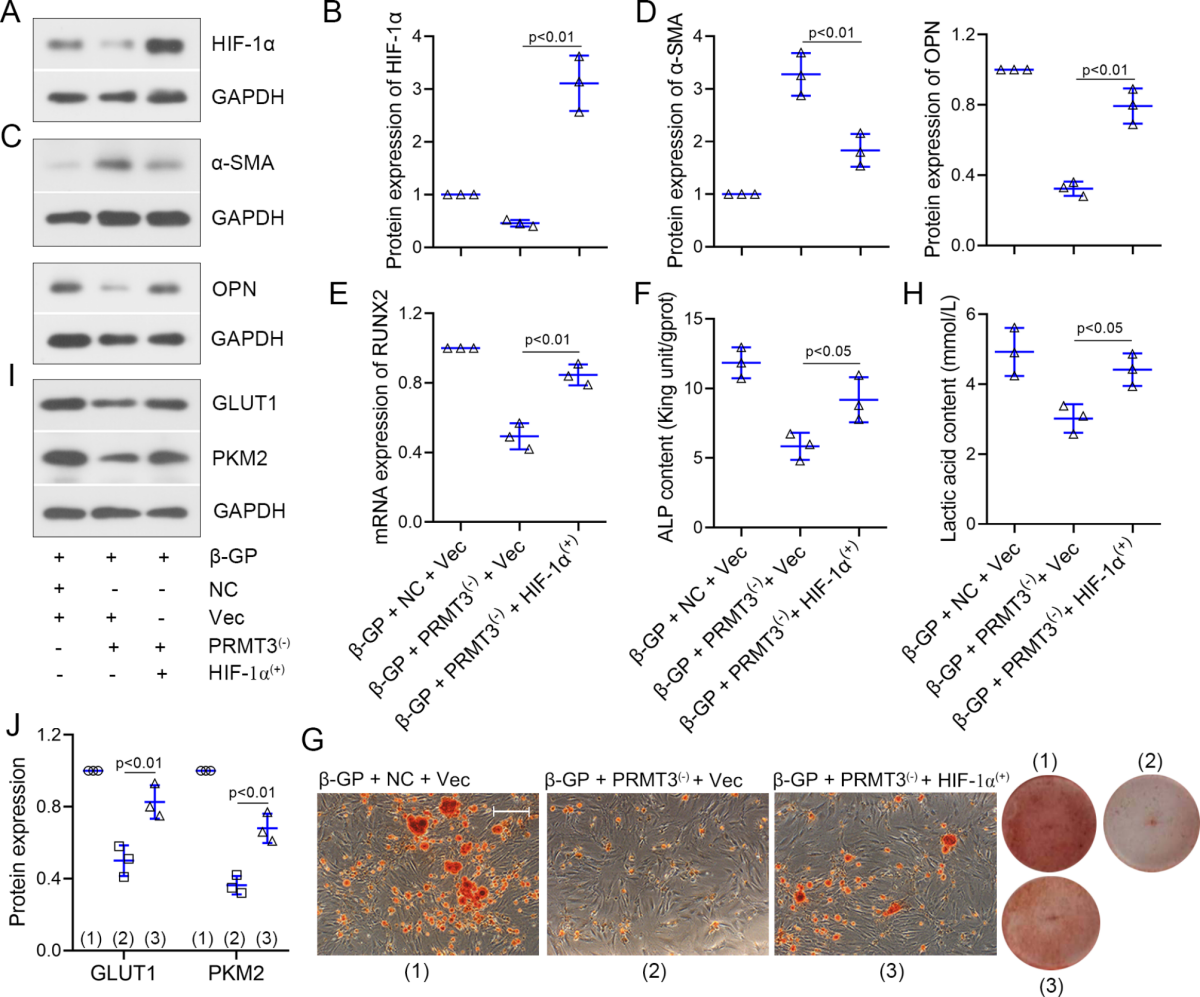
PRMT3 knockdown inhibits the β-GP-induced calcification and glycolysis by the downregualtion of HIF-1α in VSMCs VSMCs were co-infected with adenovirus Ad-shPRMT3/Ad-NCshRNA (shown as PRMT3^(−)^/NC) and Ad-HIF-1α/Vector (shown as HIF-1α^(+)^/Vec). Next, cells were incubated with 10 mM β-GP. **(A-D)** Protein levels of HIF-1α, α-SMA, and OPN in VSMCs. **(E)** mRNA expression of RUNX2. **(F)** ALP content. **(G)** Alizarin Red S staining was performed to evaluate the calcification of VSMCs. Scale bar is 100 μm. **(H)** Lactic acid content. **(I-J)** Protein expression of GLUT1 and PKM2. Data is represented as mean ± SD. p < 0.05, p < 0.01 vs. β-GP + PRMT3^(−)^ + Vec. ALP: alkaline phosphatase; β-GP: β-glycerophosphate; GLUT1: glucose transporter type 1; HIF-1α: hypoxia inducible factor 1 subunit alpha; NC: negative control; PKM2: pyruvate kinase M1/2; PRMT3: protein arginine methyltransferase 3; VSMCs: vascular smooth muscle cells

To better investigate the role of HIF-1α, HIF-1α^(+)^ or Vec was infected into VSMCs with PRMT3 downregulation. Infection with HIF-1α^(+)^ reversed the downregulated expression of HIF-1α, and OPN, and the upregulated levels of α-SMA in VSMCs in the presence of PRMT3 deficiency (Fig. 5A-D). HIF-1α overexpression reverted the PRMT3 knockdown-induced downregulation of RUNX2 mRNA expression and the decrease of ALP content (Fig. 5E-F). Unsurprisingly, HIF-1α overexpression aggravated the calcium deposition (Fig. 5G). Subsequently, the effects of HIF-1α on glycolysis were explored. High expression of HIF-1α reversed the reduction of lactic acid content and the downregulation of GLUT1 and PKM2 (Fig. 5H-J). The results indicated that the inhibitory effects of PRMT3 knockdown on β-GP-induced calcification in VSMCs might be at least partly mediated by the repression of glycolysis upon the downregualtion of HIF-1α.

### PRMT3 enhances the methylation of HIF-1α

**Fig. 6:**
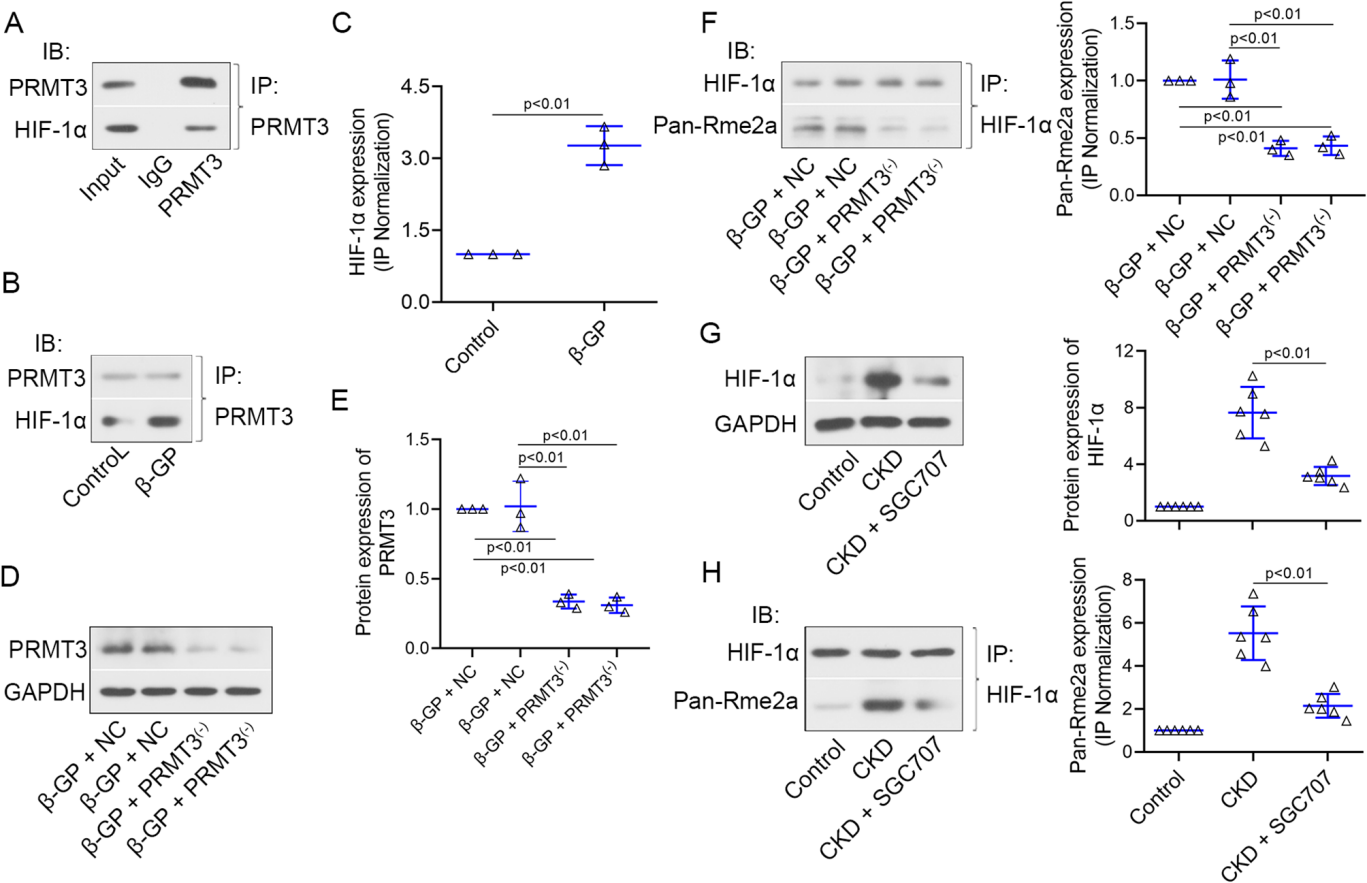
PRMT3 knockdown suppresses the methylation of HIF-1α **(A-B)** VSMCs were incubated with/without 10 mM β-GP. Whole-cell lysis was collected for co-immunoprecipitation employing anti-PRMT3 antibodies, and the samples were further subjected to western blot analysis using identified antibodies. **(C)** Quantification of HIF-1α upon IP normalization in VSMCs. **(D-E)** Protein levels of PRMT3 in VSMCs infected with Ad-shPRMT3 (shown as PRMT3^(−)^) or Ad-NC shRNA (shown as NC) in the presence of β-GP. **(F)** Whole-cell lysis was collected for co-immunoprecipitation employing anti-HIF-1α antibodies, and the samples were further subjected to western blot analysis using anti-HIF-1α and anti-Pan-Rme2a antibodies. **(G)** Protein levels of HIF-1α in aorta of mice. **(H)** Whole-tissue lysis was collected for co-immunoprecipitation employing anti-HIF-1α antibodies, and the samples were further subjected to western blot analysis using anti-HIF-1α and anti-Pan-Rme2a antibodies. Data is represented as mean ± SD. p < 0.01 vs. β-GP + NC or CKD. β-GP: β-glycerophosphate; HIF-1α: hypoxia inducible factor 1 subunit alpha; NC: negative control; PRMT3: protein arginine methyltransferase 3; VSMCs: vascular smooth muscle cells

The regulation of PRMT3 on HIF-1α expression was further explored. As shown in Fig. 6A, a physical interaction between PRMT3 and HIF-1α was observed. The interaction was enhanced in VSMCs with the β-GP induction (Fig. 6B-C). PRMT3 was downregulated in VSMCs infected with PRMT3^(−)^ in the presence of β-GP (Fig. 6D-E). PRMT3 knockdown dampened the asymmetric dimethylarginine levels of HIF-1α (Fig. 6F). Next, the molecular mechanism was testified in vivo. The CKD-induced upregulation of HIF-1α and the asymmetric dimethylarginine levels of HIF-1α were weakened by SGC707 (Fig. 6G-H). The results indicated that PRMT3 methylated HIF-1α both in vivo and in vitro.

## Discussion

Vascular calcification is an actively regulated process, which can be found in the media and intima layer of arteries. Intimal calcification usually occurs in atherosclerotic lesions, whereas medial calcification is frequently identified in type 2 diabetes and CKD (Lanzeret al. 2014). The two forms of vascular calcification sometimes coincide and overlap (Lanzeret al. 2014). Because the calcification induced by medial VSMCs is dominant in CKD progression, this study aimed to investigate the effects of PRMT3 on CKD-induced medial calcification. Herein, we induced CKD employing a two-phase diet, with the results of calcification of both media and intima layer of arteries being comparable to previous studies (Taniet al. 2017). Interestingly, the PRMT3 inhibitor SGC707 was found to mitigate the medial calcification, but not the intimal calcification according to the histopathological results. The underlying mechanism of calcification in the single layer of endothelial cells in intimal layer of arteries and VSMCs in media layer of arteries is different. The transformation of VSMCs into osteoblast- and chondrocyte-like cells is a key event in medial calcification (Tóthet al. 2022), whereas endothelial cells participate in vascular calcification via endothelial-mesenchymal transition, cytokine secretion, extracellular vesicle synthesis, angiogenesis regulation and hemodynamics (Yuanet al. 2021). It is possible that differences in pathological process might result in the different effects of PRMT3 on VSMCs and endothelial cells. It is better to explore the role of PRMT3 in endothelium, and lack of the experiments is a limitation of the manuscript. The part of experiments evoke us much interest, which will be explored in our further investigation.

Our previous main focus was on CKD induced vascular calcification, whereas the changes of renal function evokes us much attention at the present. The renal function was declined in the CKD mice, as evidenced by the elevation of the metabolic waste including serum creatinine and BUN. The changes were further reversed by SGC707 treatment. However, the effects of SGC707 on other kidney injury markers including urine biochemistries and physiological parameters as well as the injury markers in kidney remain unknown, which is another limitation of the manuscript. Subsequently, the levels of serum phosphate were detected. High levels of serum phosphate induce transformation of VSMC into osteoblast-like cells, this transformation having been closely associated to medial arterial calcification (Giachelli 2003; Lomashvili et al. 2008; Reynolds et al. 2004). Herein, there was a trend that the increased phosphorus and calcium contents in serum of CKD mice were reduced by SGC707 induction. We preferred that SGC707 exerted the protective function in vascular calcification might at least partly mediated by the reduction of phosphorus and calcium content.

PRMT3 was confirmed to be upregulated in the VSMCs in the medial artery of CKD mice by immunohistochemical staining. Interestingly, we found that VSMCs in medial artery of control mice exhibited irregular in shape, whereas VSMCs of CKD mice exhibited long shuttle in shape. We speculated that the differences between them might be associated with the changes of elastin and α-actin filaments as well as transdifferentiation of cells. Under non pathological conditions, VSMCs are arranged between elastic lamellae (Karimiet al. 2016). The elastin has oblique extension that connects with the surfaces of cells, and the smooth muscle α-actin extends obliquely across the VSMCs (Karimiet al. 2016). The crowd of abundance of elastin and α-actin might lead to the irregular appearance of VSMCs. It has been reported that elastin loss in media artery enhances the osteogenic transformation of VSMCs and is involved in medial vascular calcification (Zhouet al. 2019). Transdifferentiation of VSMCs into osteoblastic phenotype is the key cellular mechanism of medial calcification (Patelet al. 2019). It is possible that VSMCs exhibited differenced appearance between control and CKD mice due to the differences of arterial microenvironment, which is consistent with the previous study (Maet al. 2020).

Glucose metabolism is clarified to be closely linked with vascular calcification (Nakahara et al. 2017; Zhu et al. 2020). Glycolysis is involved in the osteogenic differentiation and phenotype switch of VSMCs. The inhibition of glycolysis mitigates the VSMC calcification by the regulation of PFKFB3-lactate signaling (Niuet al. 2021). The promoted effects of PRMT3 on glycolysis have been found in tumorigenesis (Leiet al. 2022), implying the potential of PRMT3 on calcification might be related to glycolysis. Herein, PRMT3 knockdown inhibited the β-GP-induced calcification of VSMCs via the repression of glycolysis. The similar role of PRMT3 loss was observed in CKD-induced vascular calcification in vivo.

Moreover, glycolysis is the major energetic source of cells that is stimulated by hypoxia. Hypoxia is a primary condition leading to vascular calcification (Baloghet al. 2019). The activation of hypoxic genes including HIF-1α is related to CKD and triggers osteogenesis (Araldiet al. 2010). Herein, β-GP induced the upregulated the expression of HIF-1α in VSMCs. Nomoto et al. have testified that HIF-1α activation contributes to elevated PFKFB3 (the key enzyme in glycolysis) levels, causing the increased glycolysis products including LDH and lactic acid in diabetes (Nomotoet al. 2020). The similar results were obtained in the current study. HIF-1α overexpression reversed the PRMT3 depletion -induced calcification and glycolysis of VSMCs in the presence of β-GP. Subsequently, the underlying mechanism of the regulation of PRMT3 on HIF-1α was explored. The expression of HIF-1α could be regulated by several posttranslational modifications including ubiquitination, acetylation, and methylation (Geng et al. 2012; Kim et al. 2016; Zhang et al. 2021). This study revealed a HIF-1α modification by methylation. Loss of PRMT3 downregulated the expression of HIF-1α both in vivo and in vitro. However, the sites of methylation HIF-1α remain unknown, which is a limitation of this study. In colorectal cancer cells, R282 of HIF-1α is asymmetrically di-methylated by PRMT3 protein, and the di-methylation of HIF-1α reduces its ploy-ubiquitination levels (Zhanget al. 2021). The similar mechanism might be existed in the progression of CKD-induced vascular calcification, which will be studied in our further investigation.

## Conclusion

**Fig. 7:**
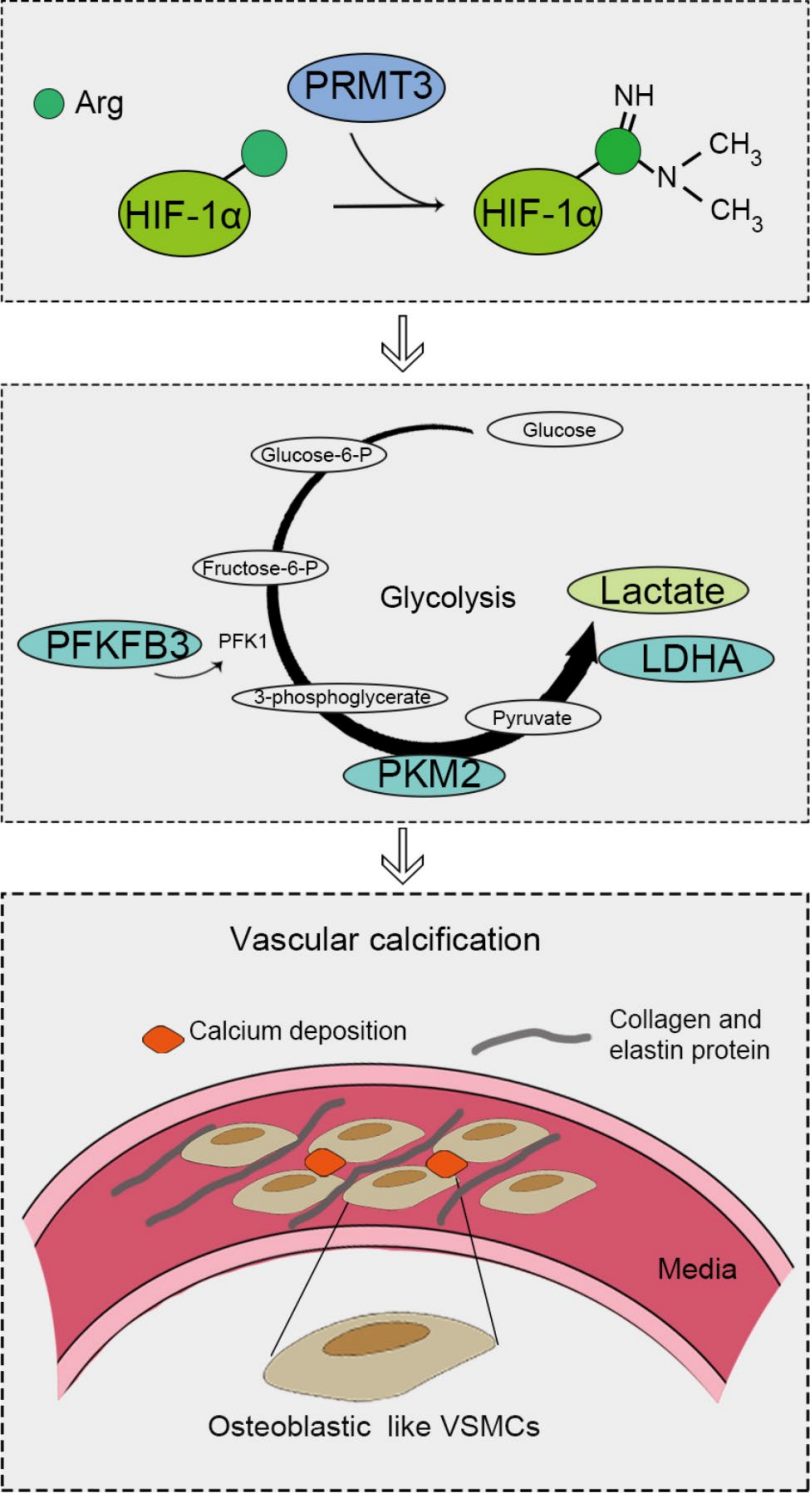
Schematic diagram of the underlying mechanism of PRMT3 on promoting the CKD-induced vascular calcification progression CKD: chronic kidney disease; HIF-1α: hypoxia inducible factor 1 subunit alpha; LDHA: lactate dehydrogenase A; PFKFB3: 6-phosphofructo-2-kinase/fructose-2,6-biphosphatase 3; PKM2: pyruvate kinase M1/2; PRMT3: protein arginine methyltransferase 3; VSMCs: vascular smooth muscle cells

The expression of PRMT3 was upregulated in VSMCs of medial artery of CKD mice and β-GP-induced VSMCs. In vivo, the inhibitor of PRMT3 (SGC707) attenuated the vascular calcification and repressed the glycolysis of CKD mice. In vitro, loss of PRMT3 alleviated the β-GP-induced osteogenic transfomation of VSMCs by the repression of glycolysis. Next, PRMT3 interacted with HIF-1α, and the knockdown of PRMT3 downregulated the protein expression of HIF-1α by inhibiting its methylation levels. Gain of HIF-1α reverted the PRMT3 depletion-induced suppression of osteogenic differentiation and glycolysis of VSMCs. The results suggest that PRMT3 boosts the CKD-induced vascular calcification by enhancing the glycolysis upon the arginine methylation of HIF-1α (Fig. 7).

## Data Availability

The data of this work is available on request to the corresponding author.

## Declarations

The manuscript does not contain clinical studies or patient data.

## Abbreviations

ALP: alkaline phosphatase
α-SMA: alpha smooth muscle actin
β-GP: β-glycerophosphate
BUN: blood urea nitrogen
CKD: chronic kidney disease
GLUT1: glucose transporter type 1
H&E: hematoxylin and eosin
HIF-1α: hypoxia inducible factor 1 subunit alpha
LDHA: lactate dehydrogenase A
NC: negative control
OPN: osteopontin
OCN: osteocalcin
PFKFB3: 6-phosphofructo-2-kinase/fructose-2,6-biphosphatase 3
PKM2: pyruvate kinase M1/2
PRMT3: protein arginine methyltransferase 3
RUNX2: RUNX family transcription factor 2
SM22α: smooth muscle 22 alpha
SMMHC: smooth muscle myosin heavy chain
VSMCs: vascular smooth muscle cells

## References

[CR1] Araldi E, Schipani E, Hypoxia (2010). HIFs and bone development. Bone.

[CR2] Bachand F, Silver PA (2004). PRMT3 is a ribosomal protein methyltransferase that affects the cellular levels of ribosomal subunits. EMBO J.

[CR3] Balogh E, Tóth A, Méhes G, Trencsényi G, Paragh G, Jeney V (2019). Hypoxia triggers osteochondrogenic differentiation of vascular smooth muscle cells in an HIF-1 (hypoxia-Inducible factor 1)-Dependent and reactive oxygen species-dependent manner. Arterioscler Thromb Vasc Biol.

[CR4] Blanc RS, Richard S (2017). Arginine methylation: the coming of Age. Mol Cell.

[CR5] Di Angelantonio E, Danesh J, Eiriksdottir G, Gudnason V (2007). Renal function and risk of coronary Heart Disease in general populations: new prospective study and systematic review. PLoS Med.

[CR6] Düsing P, Zietzer A, Goody PR, Hosen MR, Kurts C, Nickenig G (2021). Vascular pathologies in chronic Kidney Disease: pathophysiological mechanisms and novel therapeutic approaches. J Mol Med.

[CR7] Geng H, Liu Q, Xue C, David LL, Beer TM, Thomas GV (2012). HIF1α protein stability is increased by acetylation at lysine 709. J Biol Chem.

[CR8] Giachelli CM (2003). Vascular calcification: in vitro evidence for the role of inorganic phosphate. J Am Soc Nephrology: JASN.

[CR9] Hoekstra M, Nahon JE, de Jong LM, Kröner MJ, de Leeuw LR, Van Eck M (2019). Inhibition of PRMT3 activity reduces hepatic steatosis without altering Atherosclerosis susceptibility in apoE knockout mice. Biochim Biophys Acta Mol Basis Dis.

[CR10] Hwang JW, Cho Y, Bae GU, Kim SN, Kim YK (2021). Protein arginine methyltransferases: promising targets for cancer therapy. Exp Mol Med.

[CR11] Karimi A, Milewicz DM (2016). Structure of the elastin-contractile units in the thoracic aorta and how genes that cause thoracic aortic aneurysms and dissections disrupt this structure. Can J Cardiol.

[CR12] Kierans SJ, Taylor CT (2021). Regulation of glycolysis by the hypoxia-inducible factor (HIF): implications for cellular physiology. J Physiol.

[CR13] Kim Y, Nam HJ, Lee J, Park DY, Kim C, Yu YS (2016). Methylation-dependent regulation of HIF-1α stability restricts retinal and tumour angiogenesis. Nat Commun.

[CR14] Lanzer P, Boehm M, Sorribas V, Thiriet M, Janzen J, Zeller T (2014). Medial vascular calcification revisited: review and perspectives. Eur Heart J.

[CR15] Lei Y, Han P, Chen Y, Wang H, Wang S, Wang M (2022). Protein arginine methyltransferase 3 promotes glycolysis and hepatocellular carcinoma growth by enhancing arginine methylation of lactate dehydrogenase A. Clin Translational Med.

[CR16] Li FL, Liu JP, Bao RX, Yan G, Feng X, Xu YP (2018). Acetylation accumulates PFKFB3 in cytoplasm to promote glycolysis and protects cells from cisplatin-induced apoptosis. Nat Commun.

[CR17] Liao Y, Luo Z, Lin Y, Chen H, Chen T, Xu L (2022). PRMT3 drives glioblastoma progression by enhancing HIF1A and glycolytic metabolism. Cell Death Dis.

[CR18] Lomashvili KA, Garg P, Narisawa S, Millan JL, O’Neill WC (2008). Upregulation of alkaline phosphatase and pyrophosphate hydrolysis: potential mechanism for uremic vascular calcification. Kidney Int.

[CR19] Ma WQ, Sun XJ, Zhu Y, Liu NF (2020). PDK4 promotes vascular calcification by interfering with autophagic activity and metabolic reprogramming. Cell Death Dis.

[CR20] McCarty MF, DiNicolantonio JJ (2014). The molecular biology and pathophysiology of vascular calcification. Postgrad Med.

[CR21] Min Z, Xiaomeng L, Zheng L, Yangge D, Xuejiao L, Longwei L (2019). Asymmetrical methyltransferase PRMT3 regulates human mesenchymal stem cell osteogenesis via miR-3648. Cell Death Dis.

[CR22] Nakahara T, Dweck MR, Narula N, Pisapia D, Narula J, Strauss HW (2017). Coronary artery calcification: from mechanism to Molecular Imaging. JACC Cardiovasc Imaging.

[CR23] Nelson AJ, Raggi P, Wolf M, Gold AM, Chertow GM, Roe MT (2020). Targeting vascular calcification in chronic Kidney Disease. JACC Basic to Translational Science.

[CR24] Niu J, Wu C, Zhang M, Yang Z, Liu Z, Fu F (2021). κ-opioid receptor stimulation alleviates rat vascular smooth muscle cell calcification via PFKFB3-lactate signaling. Aging.

[CR25] Nomoto H, Pei L, Montemurro C, Rosenberger M, Furterer A, Coppola G (2020). Activation of the HIF1α/PFKFB3 stress response pathway in beta cells in type 1 Diabetes. Diabetologia.

[CR26] Patel JJ, Bourne LE, Davies BK, Arnett TR, MacRae VE, Wheeler-Jones CP (2019). Differing calcification processes in cultured vascular smooth muscle cells and osteoblasts. Exp Cell Res.

[CR27] Reynolds JL, Joannides AJ, Skepper JN, McNair R, Schurgers LJ, Proudfoot D (2004). Human vascular smooth muscle cells undergo vesicle-mediated calcification in response to changes in extracellular calcium and phosphate concentrations: a potential mechanism for accelerated vascular calcification in ESRD. J Am Soc Nephrology: JASN.

[CR28] Sarnak MJ, Levey AS, Schoolwerth AC, Coresh J, Culleton B, Hamm LL (2003). Kidney Disease as a risk factor for development of Cardiovascular Disease: a statement from the American Heart Association Councils on kidney in Cardiovascular Disease, High Blood Pressure research, clinical cardiology, and Epidemiology and Prevention. Circulation.

[CR29] Shanahan CM, Crouthamel MH, Kapustin A, Giachelli CM (2011). Arterial calcification in chronic Kidney Disease: key roles for calcium and phosphate. Circ Res.

[CR30] Shi Y, Niu Y, Yuan Y, Li K, Zhong C, Qiu Z (2023). PRMT3-mediated arginine methylation of IGF2BP1 promotes oxaliplatin resistance in Liver cancer. Nat Commun.

[CR31] Swiercz R, Person MD, Bedford MT (2005). Ribosomal protein S2 is a substrate for mammalian PRMT3 (protein arginine methyltransferase 3). Biochem J.

[CR32] Tani T, Orimo H, Shimizu A, Tsuruoka S (2017). Development of a novel chronic Kidney Disease mouse model to evaluate the progression of hyperphosphatemia and associated mineral bone Disease. Sci Rep.

[CR33] Tóth A, Csiki DM, Nagy B, Balogh E, Lente G, Ababneh H (2022). Daprodustat accelerates high phosphate-Induced Calcification through the activation of HIF-1 signaling. Front Pharmacol.

[CR34] Wang S, Yu H, Gao J, Chen J, He P, Zhong H (2022). PALMD regulates aortic valve calcification via altered glycolysis and NF-κB-mediated inflammation. J Biol Chem.

[CR35] Webster AC, Nagler EV, Morton RL, Masson P (2017). Chronic Kidney Disease Lancet.

[CR36] Yang L, Gao L, Nickel T, Yang J, Zhou J, Gilbertsen A (2017). Lactate promotes synthetic phenotype in vascular smooth muscle cells. Circ Res.

[CR37] Yuan C, Ni L, Zhang C, Hu X, Wu X (2021). Vascular calcification: new insights into endothelial cells. Microvasc Res.

[CR38] Zhang X, Wang K, Feng X, Wang J, Chu Y, Jia C (2021). PRMT3 promotes tumorigenesis by methylating and stabilizing HIF1α in Colorectal cancer. Cell Death Dis.

[CR39] Zhou YB, Zhou H, Li L, Kang Y, Cao X, Wu ZY et al. Hydrogen Sulfide prevents elastin loss and attenuates Calcification Induced by High glucose in smooth muscle cells through suppression of Stat3/Cathepsin S Signaling Pathway. Int J Mol Sci. 2019;20(17). 10.3390/ijms20174202 PMC6747320 31461977

[CR40] Zhu Y, Han XQ, Sun XJ, Yang R, Ma WQ, Liu NF (2020). Lactate accelerates vascular calcification through NR4A1-regulated mitochondrial fission and BNIP3-related mitophagy. Apoptosis.

[CR41] Zou Y, Webb K, Perna AD, Zhang Q, Clarke S, Wang Y (2007). A mass spectrometric study on the in vitro methylation of HMGA1a and HMGA1b proteins by PRMTs: methylation specificity, the effect of binding to AT-rich duplex DNA, and the effect of C-terminal phosphorylation. Biochemistry.

